# Nutrition among children of migrant construction workers in Ahmedabad, India

**DOI:** 10.1186/s12939-019-1034-y

**Published:** 2019-09-17

**Authors:** Divya Ravindranath, Jean-Francois Trani, Lora Iannotti

**Affiliations:** 1 Indian Institute for Human Settlements, No. 197/36, 2nd Main Road, Sadashivanagar, Bengaluru, 560 080 India; 2 Brown School, Washington University, St. Louis, USA

**Keywords:** Migrant children, Malnutrition, Construction workers, Informal work

## Abstract

**Background:**

Millions of poor households in India undertake short duration rural to urban migration along with their children to find work in the informal economy in the city. While literature has documented the precarity of such temporary jobs, typically characterized by low wages, insecure jobs, harsh recruitment regimes and economic vulnerability, little is known about its implications for children who migrate with their parents to the city. In this study, we draw attention to children of migrant construction workers and focus on their overall nutritional well-being, which remains under-studied. Our objectives were to categorize the current nutritional status of children under the age of five and determine the underlying causes of poor nutritional outcomes.

**Methods:**

The field work for this study was undertaken between May 2017 and January 2018 at five construction sites in the city of Ahmedabad. We undertook anthropometric measurements of children under the age of five [*N* = 131; (male: 46%, female 53%); (mean age: 31.7 months)] and categorized their nutritional status. Using the UNICEF framework on undernutrition, we examined the underlying causes of poor nutritional outcomes among this group of children with the help of qualitative methods such as interviews, focused group discussions and participant observation at the field sites.

**Results:**

Undernutrition was highly prevalent among the children (*N* = 131): stunted (40.5%); wasted (22.1%); and underweight (50.4%). We found common factors across parents interviewed such as similar misperceptions of malnutrition, long hours of work and lack of childcare provision at the worksite which resulted in disrupted quality of care. While socio-cultural beliefs and lack of information influenced breastfeeding, other factors such as inability to take breaks or lack of space further impaired infant feeding practices more broadly. Lack of dietary diversity at home, poor hygiene and sanitation, and economic inability to seek healthcare further affected child nutritional status.

**Conclusions:**

Our study addresses a critical gap in migration literature in India by building a comprehensive narrative of migrant children’s experiences at construction sites. We find that parents’ informal work setting exposes children to a nutritionally challenging environment. Policies and programs seeking to address undernutrition, a critical challenge in India, must pay attention to the specific needs of migrant children.

## Introduction

Short-duration rural to urban migration is an important livelihood strategy for millions of poor households in India. These short-term^1^ migration streams are dominated by historically marginalized social groups such as the Scheduled Tribes (ST) and the Scheduled Castes (SC) that also often experience chronic poverty [1]. In the city, migrant households mostly engage in temporary work that are characterized by low wages, insecure jobs, harsh recruitment regimes and economic vulnerability, which are known to be typical of informal work environments [2, 3]. A crucial aspect of labour migration in India is that many migrant households move with their entire families including children^2^ [4, 5]. Previous research in India has shown that even though migration provides economic opportunities to households, migrant children face several disadvantages in the destination region. For instance, migrant children experience poorer educational outcomes as their access to education is constantly disrupted by frequent spells of migration [5–7]. Similarly, children of seasonal or short-duration migrants become vulnerable to nutrition insecurity as they lack easy access to subsidized food and fair worse than non-migrants on most health indicators [8]. Several studies have also documented that the uptake of immunization is low among migrants [9] because of lack of awareness, time constraints, high costs incurred for vaccination and difficulty in traveling to health centres in urban areas [10–13]. As children stay unattended at their parents worksites, exposed to unhygienic living conditions and unsafe environment there are large concerns about their safety, and overall health and wellbeing [4, 14]. While these studies provide critical insights on the experiences of migrant children in the city, an in-depth analysis of the link between migration, parents’ informal work environment and nutritional wellbeing remains underexplored. This study examined nutrition among one particular group -children of migrant construction workers, who live on construction sites with their parents. Our objectives were to categorize the current nutritional status of children under the age of five and determine the underlying causes of poor nutritional outcomes among these children.

### Children at construction sites

The construction sector, which employs close to 50 million people, is heavily dependent on migrant workers. A large proportion of workers (approximately 93%) in the construction sector undertake informal work, which implies the absence of social and legal protection [15]. Migrant workers experience exploitative labour arrangements as they work under hazardous conditions, with little bargaining power [7, 16]. Labourers are employed through a complex network of contractors and sub-contractors, who take little responsibility for the work and living environment, and commonly engage in wage exploitation [2]. Construction work is considered conducive to family-based migration as it provides work to male and female members of a family. When women migrate, they tend to bring their children along, especially when the child is younger and is in need of care [3]. While one can witness large presence of children at most construction sites, there has been no official exercise to enumerate them. Here, we focus on this context of migration and informal work environment to understand how it shapes children’s nutritional status.

### Conceptual framework for the study

**Fig. 1:**
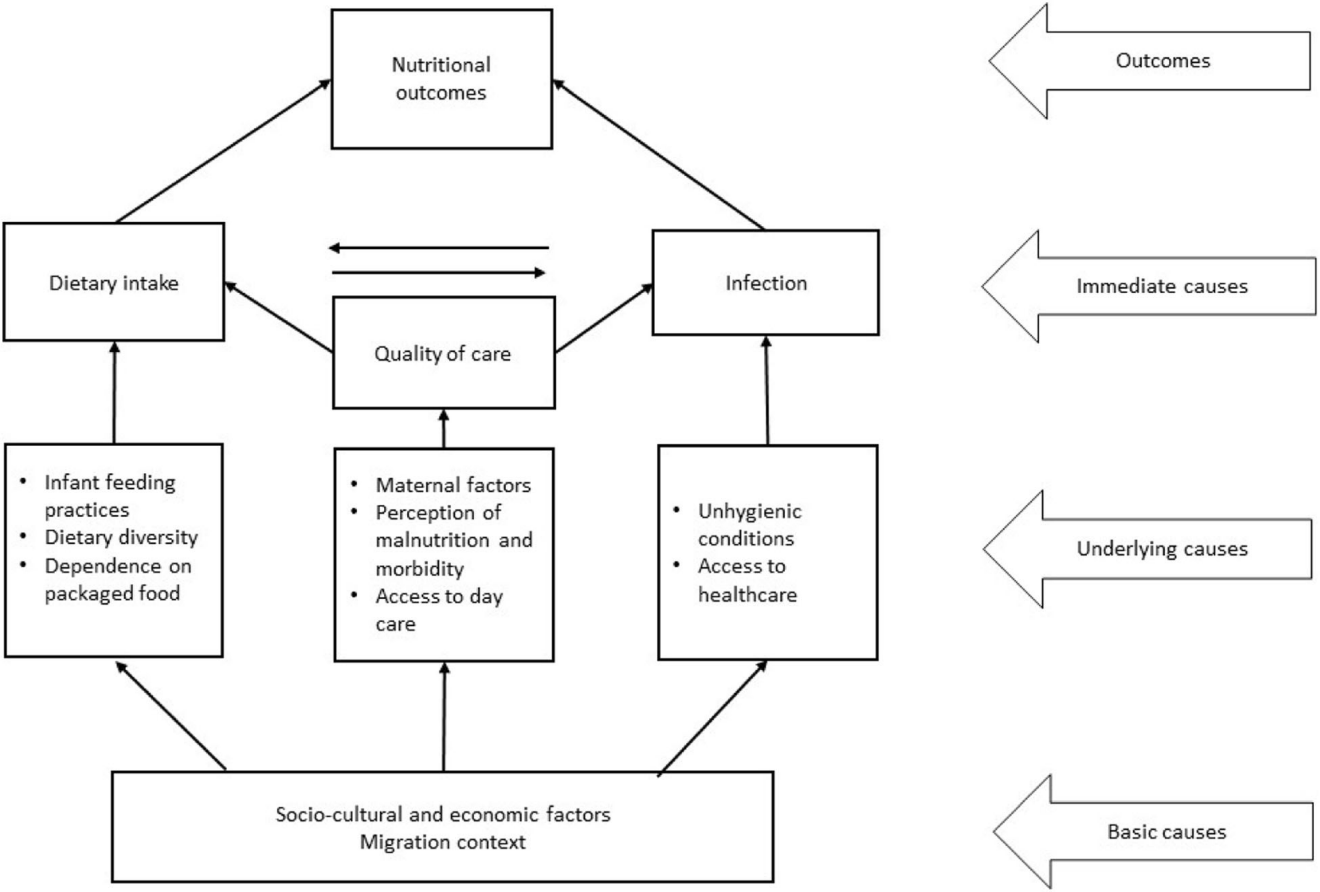
Conceptual Framework. Adapted from UNICEF conceptual framework on undernutrition [26]

In this study we specifically focus on undernutrition among children of migrant construction workers^3^. Extant literature has established that undernutrition has long term (morbidity and mortality, chronic diseases, cognitive and physical impairment among others) as well as short term consequences (reduced immunity and susceptibility to infections, metabolic alteration etc.) for children [17–20]. Research has also found that undernutrition affects physical and cognitive development, schooling and productivity [21, 22]. Researchers note that poor nutrition in childhood lowers the ability to perform well in school, and in later years at work [23]. Reduced efficiency means sub-optimal income opportunities in adulthood. This impacts households’ or individuals’ economic status, as well as country’s economic potential [24]. Black et al. (2013, p.373) [25] estimate that undernutrition reduces “a nation’s economic advancement by at least 8% (direct productivity losses, losses via poorer cognition and losses via reduced schooling).” The widely-used UNICEF framework on undernutrition (Fig. 1) provides a broad understanding of the various causes of undernutrition. It identifies three main categories of determinants of childhood undernutrition: the immediate causes, the underlying causes and the basic causes. Our focus in the study was on the underlying causes, broadly classified into poor dietary intake, inadequate care practices and disease environment.

## Methods

This cross-sectional, mixed methods study was undertaken in the city of Ahmedabad, located in the western state of Gujarat, India. Ahmedabad is experiencing rapid urban expansion in the city, and the construction sector attracts migrant workers from various parts of the country [27]. The field work for this study was conducted between May 2017 and January 2018 at five construction sites in the city. Access to these construction sites was provided by two partner organizations that run day-care facilities for workers’ children.

### Data collection

Table 1 provides a snapshot of the study sample, sampling criteria and methods adopted to collect data with each of these groups.

**Table 1:** Sample, sampling criteria and methods

Sample	Sampling criteria	Methods
Children (*N* = 131)	Criterion sampling (children under the age of five)	Anthropometric measurements; Observation
Mothers (*N* = 50)	Criterion sampling (mothers with at least one child under the age of five)	Indepth interviews; Anthropometric measurements; Observation
Fathers (group 1: *N* = 10; group 2: *N* = 8)	Criterion sampling (fathers with at least one child under the age of five)	Focus group discussions
Other stakeholders (*N* = 13)	Snowball sampling (stakeholders working with migrant communities)	Semi-structured interviews

We adopted criterion sampling [28] based on age to enrol children in our study. All children under the age of 5 years who were present at the study site were assessed for eligibility, recruited, and enrolled in the study. In all, 131 children (male: 46%, female 53%); (mean age: 31.7 months) participated in the study. In 35% cases (*N* = 46), we used a local calendar to arrive at an approximate age of the child as the parents were unable to report exact age. While this calendar was useful in recalling events of the immediate past, it may have led to some age heaping around local festivals in the past years. Since the WHO Anthro software uses exact birthdate of the child, we have also reported results for a truncated sample that excludes this 35% of the sample. The study team (comprised of the first author of this paper and a research assistant) followed a globally standardized protocol for measuring anthropometry [29]. In case of a difference in the first two measurements (0.5 cm for height and 100 g for weight), a third measurement was undertaken by the team. The anthropometric measurements were collected using Seca 417 for recumbent position (length) and Seca 213 for standing position (height) and Seca 876 for weight (Seca, Hamburg, Germany). We also engaged in participant observation [30] to study children’s interaction with their environment and documented various aspects of their everyday lives.

The criterion for mothers and fathers in the sample was that they should have had at least one child below age five. We approached all mothers whose children’s anthropometric measurements were undertaken to participate in the study. A sub-sample of mothers (*N* = 50) agreed to be interviewed for this study. The others either could not give us time during our field work period or had migrated out within that time frame. The final sample included 89 mother-child dyads (mother paired as a dyad with each of her children). Mothers’ sample included six pregnant women and 29 lactating mothers. In-depth interviews were conducted with the women either in the morning at the labour colony where families lived or in the day care centre during lunch break. As mothers are the primary care givers in this context, our interviews focused on themes such as care practices, dietary intake, living conditions and access to healthcare services. We also undertook anthropometric (height and weight) measurements of mothers, which were used to calculate their BMI status.

In order to capture the perceptions of fathers, two Focus Group Discussions [Group 1 (*N* = 10), Group 2 (*N* = 8)] were conducted with men who had at least one child under the age of five. The FGDs focused on migration patterns, income utilization, and access to food and healthcare. Through snowball sampling [31], we also reached out to other stakeholders (*N* = 13) – construction union leaders, staff of not for profit organizations, maternal health activists, doctors and academic researchers working on health or migration and government employees. Semi-structured interviews were undertaken with this group of participants, which focused on the larger context. By applying various methods of data collection (observation, interviews and focus group discussions) and by reaching out to various types of respondent groups as shown in Table 1 (mothers, fathers and other stakeholders) we were able to triangulate our data, which helped us to achieve a more complete understanding of the issues under study [32].

### Data analysis

The anthropometric measurements were used to calculate z-scores of height-for-age (HAZ), weight-for-height (WHZ), and weight-for-age (WAZ), with the help of the World Health Organization’s software ‘Anthro’ to categorize nutritional status as stunted, wasted or underweight respectively among children who were two standard deviations below the reference population. The interviews and FGDs were conducted in Hindi and Gujarati, languages spoken by the construction workers, and translated and transcribed into English. The first author and her research assistant are familiar with both the languages respectively, and thus were able to assess the accuracy of the translated transcripts.^4^ Observational notes were also coded and analysed. Using MAXQDA, we employed a combination of a priori coding and emergent coding. A priori codes (example: the sub-codes of underlying causes included living conditions, feeding practices, access to healthcare) were created beforehand based on our understanding of the literature. Emergent codes (example: lack of vaccination records, perception of child nutrition status) were drawn from the text. The emergent codes were then classified under various underlying causes. This dual approach enabled us to undertake constant iterations to develop, refine and modify questions for newer information [33]. We also continually tested our data and its interpretations by periodically discussing our findings with staff members of partner organizations as well as with the members of the community in informal discussions to address misrepresentation of the data [34].

## Findings

### Sample description

In our study sample, as shown in Table 2, a majority of children came from districts within the states of Gujarat and Rajasthan in Western India. Less than half hailed from districts in Madhya Pradesh and Chhattisgarh in Central India, and the remaining children were from Bihar and West Bengal in Eastern India (See Table 2). In terms of caste, the largest group of children were from Scheduled Tribes (ST), while the others belonged to Scheduled Castes (SC) and Other Backward Classes (OBC). Over half the children in our sample were females.

**Table 2:** Demographic profile of children in the sample (*N* = 131)

		N	%
Source regions (state and district)	Gujarat		
	Dohad	14	10
	Panchmahal	15	11
	Rajasthan		
	Dungarpur	19	15
	Banswara	12	9
	Madhya Pradesh		
	Jhabua	20	16
	Satna	3	2
	Chattisgarh		
	Jhanjgir Champa	22	17
	Bihar		
	Kathiar	10	8
	East Champaran	5	4
	West Bengal		
	Cooch Bihar	3	2
	Malda	3	2
	Bardhaman	4	3
	Dakshin Dinajpur	1	1
Caste	Scheduled Tribes	105	80
	Scheduled Caste	15	12
	Other Backward Classes	11	8
Sex	Female	70	54
	Male	61	46

### Household factors

Approximately 70% of the mothers we interviewed for the study reported that their households undertook seasonal migration. They returned to their villages periodically for agricultural work, weddings, festivals, and other cultural events. The other group of women reported that their households had been living away from home for a longer duration – ranging from 1 year to 5 years. These semi-permanent migrant households had previously migrated to other cities as well. In general, all migrant households reported residing at multiple locations within the city, as they moved from one construction site to another at the end of each project cycle. The duration of stay at each site usually spanned between 3 months to over a year based on the size of the project. In terms of wage patterns, mothers received between Rs. 250–300 (minimum wage is Rs.306 per day). However, wages were usually paid to the family as a unit and were collected by the male head of the family. It was common practice for households to take *kharchi –* a form of cash advance (from their own monthly wages), to meet weekly expense of food, medical care, remittances and debt repayments. While the system of *kharchi* provided workers liquid cash for regular expenses, it also increased workers’ wage insecurity and dependence on the contractor for constant economic support. In the latter sections we explain how migration and wage patterns affect households and children’s health in particular.

### Nutritional status of children

Half the children in our study sample were underweight, slightly less than half were stunted and one in five experienced wasting. The prevalence of wasting and underweight was higher in the age strata 6–11 months and 48–60 months. All three indicators were the lowest in the 36–47 months age strata (See Table 3). There was no statistical difference in stunting, wasting and underweight in the sample by sex.

**Table 3:** Standardized indicators of anthropometry among children (under 5) in the study sample based on the World Health Organization growth standards (2006)

Age in months	Mean anthropometric Z scores (SD)			Prevalence of undernutrition (%)		
	HAZ	WAZ	WHZ	Stunting	Underweight	Wasting
0–5 (*N* = 7)	−1.66 (1.28)	−1.94 (0.78)	−0.98 (1.02)	42.9	42.9	14.3
6–11 (*N* = 15)	−1.79 (1.05)	−2.17 (1.08)	−1.45 (1.03)	46.7	53.3	33.3
12–23 (*N* = 30)	−1.60 (1.16)	−1.75 (0.93)	− 1.36 (1.05)	46.7	46.7	20
24–35 (*N* = 25)	−1.74 (0.59)	−1.99 (0.47)	− 1.46 (0.61)	40	52	8
36–47 (*N* = 18)	− 1.74 (0.85)	− 1.70 (0.80)	− 1.01 (0.72)	27.8	33.3	5.6
48–60 (*N* = 36)	−1.73 (0.83)	−2.05 (0.93)	− 1.51 (1.19)	38.9	61.1	38.9
All age groups (*N* = 131)	−1.71 (0.92)	− 1.93 (0.86)	− 1.36 (0.98)	40.5	50.4	22.1
Female (*N* = 71)	−1.62 (0.94)	−1.86 (0.88)	− 1.29 (1.02)	52.8	51.5	51.7
Male (*N* = 60)	−1.79 (0.89)	−2.00 (0.83)	−1.44 (0.92)	47.2	48.5	48.3
Truncated sample^a^ (*N* = 85)	−1.72 (0.95)	−1.89 (0.91)	− 1.30 (0.99)	38.8	49.4	18.8

^a^This excludes 35% of the sample whose birthdates were calculated based on the local calendar

### Underlying causes of undernutrition

Based on the conceptual framework presented above, in this section we document various factors that contribute to the underlying causes of undernutrition.

### Maternal factors

As seen in Table 4, a majority of mothers (66%) in our sample did not know their age. Similarly, most mothers (72%) noted that they had never attended school. We found a positive (coeff = .28), but statistically nonsignificant relationship between mothers’ schooling and child stunting in our sample. Over half of the mothers were underweight or suffered from low BMI (M = 18.22, SD = 1.7)*.* We found a statistically significant positive correlation between mothers’ BMI and child stunting (coeff = 0.6) and underweight (coeff = 0.5).

**Table 4:** Maternal factors (*N* = 50)

		N	%
Age (rough estimates)	Between 15 and 20	9	18
	Between 20 and 25	8	16
	Don’t know	33	66
Education level	No schooling	36	72
	Some Schooling	14	28
	Up to class 4	6	12
	Up to class 8	8	16
BMI status (kg/m2)	18.50–24.99	24	48
	17.00–18.49	13	26
	16.00–16.99	8	16
	< 16.00	5	10

### Perception of nutrition and morbidity

When we asked mothers if their child was well nourished, all mothers except four replied in the affirmative. One of the mothers who thought her child was malnourished attributed it to her son’s low birth weight:

*He was very small at birth… yes, doctor told us 1.5 kg. We went to Siliguri and stayed in the hospital for ten days. He is better, but always sick.*

Most mothers, however, reported that their children fell ill very frequently. Diarrhoea, cold, cough and fever were cited as the most common ailments. These illnesses were mostly associated with lack of clean drinking water or change in weather, as suggested by this mother:

*It’s* (sniffling nose) *because it’s very cold these days. He will be fine in a few weeks.*

A father thought that poor water quality caused frequent illnesses.

*The water is so dirty here. Even we fall ill, children are going to be more affected.*

According to a day care staff member, parents were rarely able to recognize undernutrition among their children.

*Most parents don’t know their children are malnourished. We measure them here and tell parents that something has to be done soon. But our window of opportunity is also very small. We give them counselling and feed them healthy food, and just as we are seeing some improvement, they leave the city or go to another site. Though I can’t say with certainty for each case, our experience has been that they generally go back to being very malnourished.*

### Access to and perceptions about day care facilities

All children included in this study sample had access to day care facilities, which provided them food, clean drinking water, access to a toilet, as well as a safe space to play and learn. Parents were conscious of the usefulness of this service. For instance, one mother suggested:

*In the village there are so many people to look after our children when we go to work. Here there is no one, so the school* (day care) *is good.*

At one construction site, the day care staff member mentioned that most parents liked to send their children to the day care because the worksite was far from the labour colony, and the parents found it difficult to keep a check on their children:

*Parents appreciate the day care. Mothers don’t have the time to cook. I think it’s nice for them to know their children are eating good food here and are safe.*

Mothers who felt they had benefitted from the services also complained that day care facilities were rarely available on other construction sites, as noted below:

*When there is no care, we take the child with us to the site… it’s dangerous. But what can we do?*

*We are always worried that children will hurt themselves. We tell them, but they are children, they don’t always listen. Its good we can leave them here*

### Infant feeding practices

Over three-fourths of the mothers interviewed for this study reported breastfeeding their children since birth. The frequency of breastfeeding ranged between 2 and 6 times in a day. Women reported several barriers to breastfeeding at the construction site. Inability to take regular breaks was considered to be a major concern for most women, as articulated by one mother:

*When we are at work, we cannot come often to feed the child. The contractor doesn’t allow us.*

Some women said that they tried to take their children to the construction site for frequent feeding but were not comfortable breastfeeding in front of others, as shared by this mother:

*But I don’t like feeding her there* (workplace)*. Everybody is watching. It doesn’t feel good. So, I try to come back every few hours, but it’s not possible every day. Sometimes we are working very far away.*

Though breastmilk was the chief source of diet for infants, most mothers reported giving their children water or water mixed with sugar, especially in summer to beat the heat. An infant’s mother noted:

*Milk is not enough when it is so hot. You have to give something more otherwise the child is always crying.*

Some mothers also introduced diluted cow’s milk or milk powder mixed in water to their children’s diet within the first 6 months as suggested below:

*We give cow’s milk from the packet in the morning*… *and sometimes at night* … *few spoons mixed with water.*

A doctor we interviewed for this study told us that giving water or diluted milk, even honey, to infants was common practice in households. He added:

*Sometimes the mothers leave the child with the older sibling, and when he or she is crying unendingly, the sibling is forced to give water. How much can we expect from an eight or ten-year-old child looking after a baby?*

Among those women who could not breastfeed their children, the commonly cited reason was *‘not enough milk’*. A mother noted:

*I gave my child lactogen since birth… it’s expensive but when I don’t have milk I do that… doctor* (in the city) *told me about it*

The nurse at the day care noted that in her experience first time mothers were more likely to believe they had insufficient milk:

*When first-time mothers are away from their families there is no passing of traditional wisdom that can help them to look after a child. And of course, they don’t receive formal counselling either, they are often confused. We tell them that the breastmilk they have is sufficient for the child. But they are always concerned that they don’t have enough milk*

In some cases, however, breastmilk continued to remain the mainstay in the child’s diet even after the six-month mark. A mother claimed that her last child almost entirely depended on breastmilk until he was 3 years old.

*My child doesn’t eat anything else, so I still breastfeed him…it’s not enough, but what can I do?*

When we asked the doctor, what derailed the transition out of breastfeeding he responded:

*It’s easy for us to say start complementary feeding at six months. But for the mother it’s better to keep the child totally dependent on breastmilk than cook separately some sort of semi-sold food that the child can eat. Once the child has teeth and can chew food it’s easier. But that transitionary phase between breastfeeding and full meal, essentially the complementary feeding phase is often skipped.*

### Dietary diversity

During our home visits, we observed that the meals in the households comprised of a combination of two of the following items: *dal* (lentil), *chawal* (rice) or *roti* (flat bread) made of corn/wheat flour, *sabzi* (vegetable curry) usually made out of tomatoes, onions and eggplants that were easy to clean and cook. None of the households reported the consumption of fruits, eggs or leafy greens. Only four mothers reported cooking meat in the past month. Several migrant households carried maize flour, wheat flour and a few other non-perishable items that were grown in their villages. These proved to be the dietary mainstay of the family, which had to be substituted by locally available products at the end of the stock. A large majority of households complained that the cost of food items was much higher in the city, which prevented them from consuming greater variety of food. A father’s comment below reflects this:

*It's* (milk) *available in the shop. But it costs more. For our three children we need one full packet. In the village we have a cow at home so we don’t spend that much.*

Another mother shared similar views:

*We try to buy what we can. But it costs a lot of money.*

All children at the day care centres received breakfast, lunch and an evening snack. When we asked mothers about their children’s food intake at home most mothers complained that their children did not eat well at home, as seen below:

*My child doesn’t eat much. Little dal and rice, if I force him a lot.*

A father attributed low food intake among children to the overall environment:

*If children are not happy how will they eat. In the village they are free and that makes them eat better. But what can we do. We have to come here.*

### Packaged food

All mothers reported that their children regularly consumed packaged food. We also observed that when mothers came to drop children off at the day care, they often handed out packaged potato chips, cookies (locally called biscuits) or other fried items that were sourced from shops in the vicinity. One mother told us that it was convenient to give her child fried snacks:

*He cries for it every day. If he is hungry I have to give him something so I give him this (showing a packet) till I finish cooking.*

A day care staff member noted that this was a major cause of concern, and was often discussed with parents:

*It’s the easiest way to calm him* (child) *down… Though it seems like only Rs. 5 to 10 a day, it is actually a big expense overtime, especially if there are more kids in the family. We are trying to encourage them to buy milk with that money. But these things are available just outside the construction site, for milk they have to go further away.*

The doctor noted that we had to look at this issue in the broader context:

*I am not sure we can blame the mothers for this. When she has done heavy laborious tasks for 10-12 hours a day… how do we expect her to go back home and make sure her child is eating well? We need to look at the supply side deficit in healthy packaged food*

### Hygiene conditions

We observed that the temporary housing structures in labour colonies were built in unused parts of the construction site, too close to a dump yard as we saw in one case or next to scrap materials. At one of the construction sites we visited, the labour colony was built on undulated land, which flooded during rains. Though there were toilets in the labour colonies, parents did not always encourage children to use them either because they were very crowded or not well-maintained. As a result, children especially the younger ones, defecated in the open right next to their house where the mother could clean easily. A mother explained:

*The toilets are there. But children go here. He* (son) *is scared to go alone and I can’t go every time.*

Similarly, access to portable water was a cause of concern. At two labour camps, we observed that parents brought home drinking water from an uncovered tank that was used for bathing and washing. One mother remarked:

*We always find insects in the water. What can we do? We drink this only.*

A day care worker told us that though there were regular counselling sessions parents found it difficult to practice basic hygiene.

*We teach the children to wash their hands before every meal or after using the toilet* (at the day care)*. But what is the use of washing at the labor camp, the water is so dirty there. We also tell them to have a bath every day, but parents leave so early and come so late, it’s difficult for them. Children fall ill every week because of these things.*

### Access to healthcare

Households had to locate private medical care services every time they moved within the city. They found localized services more convenient in terms of timings and distance, than government run hospitals that were not always located in their immediate vicinity. A mother who worked at a construction site at the periphery of the city noted:

*It is far* (government hospital). *It was taking us full day. The lines are very long there for everything. They make us wait to even get what is in the parchi* (prescription).

In addition, the popular belief among parents was that private hospitals provided “*better*” medical care than government hospitals. As a result, when a child fell ill s/he was taken to the nearest private medical facility. However, parents also noted that because private medical care was more expensive they tried to delay hospital visits until it was absolutely necessary. A father noted:

*We go when the child is very sick. Otherwise we don’t. The doctor gives one glucose bottle. Every trip costs us Rs. 500 or more*

In most cases the families did not have a vaccination record either because it was never provided to them or because they had left it behind in the village. Consequently, parents did not follow up on vaccination as suggested by this mother:

*When he* (son) *was born the doctor gave him all the injections. We haven’t gone to the doctor after that.*

Two families that had a complete record of the child’s vaccination history reported that cost of vaccines was a major constraint. In this context the mother said:

*We got some* (vaccines)*. We will organize money and get another soon.*

## Discussion

Though India has made progress in addressing undernutrition over the last decade, the pace of change has been insufficient to meet the global targets (2025) to improve maternal, infant and young child nutrition [35]. The National Family Health Survey (NFHS 2015–16) report shows that the prevalence of stunting (38.4%), wasting (21%) and underweight (35.7%) continues to remain high in the country [36]. The prevalence is highest among SC and ST groups [37, 38]. Anthropometric data from our study, which is also dominated by these social groups, suggested that while the proportion of stunting (40.5%) and wasting (22.1%) among migrant children in our sample were close to the national average, the proportion of underweight children (50.4%) was considerably higher. Strikingly, despite the high proportion of undernutrition among children in our study sample, an overwhelmingly large number of mothers did not consider their children to be malnourished. Research from various settings has shown that parents’ (especially mothers’) perceptions of child undernutrition are likely to influence provision of care, and children’s overall growth and development are associated with households time spent in caring for children [39, 40]. However, mothers from poor households often lack appropriate information on aspects such as optimal feeding practices or need for extensive care during illnesses which are critical for addressing undernutrition [41, 42]. Migrant women at construction sites experienced tremendous time constraints as they undertook domestic work and simultaneously engaged in long hours of construction work. In addition, they lacked social support in the city, which was more easily available in the village in the form of other family members. While in this case there was access to day care services, mothers also noted that at other work sites these services were missing, which further impaired their ability to provide care.

With reference to other maternal factors, in our study a majority of mothers had never attended school. Literature suggests that maternal education has an effect on child development, such that children of mothers who have achieved higher education experience better health outcomes than those with lower or no education [43–46]. Similarly, it is also known that mother’s undernutrition contributes to child undernutrition and poor health outcomes [47]. This aspect was reaffirmed in our study where we found an association between child anthropometry and mother’s BMI status.

An important aspect of infant care is optimal feeding practices. Exclusive breastfeeding for the first 6 months is known to be associated with reduced mortality and morbidity. Children who are exclusively breastfed for the first 6 monthss of life are more likely to achieve optimal growth, nutrition and development and are less likely to be underweight [48]. In the literature non-exclusive breastfeeding practices have been attributed to socio-cultural beliefs, misconceptions, lack of information and perception of insufficient milk [49, 50]. While these aspects find resonance in our study as well, with women providing top feed in the form of water, diluted milk and water with sugar within the first 6 months, in this case breastfeeding practices were also affected by the constraints imposed by the mothers’ work environment. Women in our study noted that they were unable to take regular breaks from work, found it difficult to shuttle between the work space and home, lacked separate spaces for breastfeeding, and felt uncomfortable feeding in front of others at the worksite. These constraints caused by an informal work environment reduced their ability to breastfeed frequently. It also encouraged top feed, especially when the child was left in the care of an older sibling. Complementary feeding is absolutely critical from the onset of 6 months, failing which children may experience the risk of stunting [51, 52]. Research has shown that in poor households breastfeeding and complementary food practices are often far from optimal [53]. In our sample, while mothers did not practice exclusive breastfeeding within the first six months, it continued to remain the dietary mainstay for children until two or even 3 years.

Adequate dietary intake in the form of diverse food items are known to be critical for children for higher micronutrient intake and their daily energy needs [54, 55]. Children in our study sample were able to receive one full meal and two smaller meals at the day care centres, however, the overall composition of diet at home was limited and less diverse. Households did not include dairy products, variety of vegetables and fruits, pulses, coarse grains, meat and fish. Many parents attributed the lack of diversity in their diets to high cost of food items in the city, especially staple food items such as rice, wheat and local fruits and vegetables. Though diet-based strategies that include diverse food items have been pronounced the most efficient way to control micronutrient deficiency [56], it has been noted that low income and high food prices negatively influence micronutrient intake [57].

A vast body of work has shown that poor hygiene, lack of provision of water and sanitation effect children’s health and nutritional status [58–60]. The labour colonies we visited had poor hygiene and suffered from inadequate provision of water and sanitation facilities. These factors are known to contribute to intestinal infection that has greater impact on the nutritional status of children and is a common trigger for the cyclic relationship between undernutrition, infections and diseases [61]. In the case of our study, while households reported frequent bouts of illnesses among their children, their uptake of healthcare services was delayed and even remained limited because of paucity of time and economic constraints that reduced their ability to seek care. Families also reported lack of familiarity with health systems in urban areas especially when they had to locate services at a new location each time. These findings reflect similar experiences elsewhere, where households delay seeking healthcare for sick children because of distance or high out of pocket expenditure contributing further to morbidity and mortality [4, 62, 63].

### Study limitations

At the field work stage, we made several attempts to gain entry into construction sites that did not have interventions by not-for-profit organizations. However, the presence of two women (researcher and her research assistant) seeking to talk to other female workers was treated with suspicion and hostility by male security guards and contractors. After due consultation with others who have worked in this field, we restricted our sample to those sites where not-for-profit organizations were able to provide us entry and access. Since construction sites that have interventions are likely to have more informed or sensitive employers, the problems articulated by our sample may be an underestimation of the daily trials that workers and their children encounter at other construction sites. Children who are cut off from “care and security, health and nutrition, learning and exposure, and an overall normalcy of childhood” are likely to be more vulnerable [64]. We tried to overcome this by asking mothers questions that spanned their experiences over multiple sites and migration trips.

## Conclusion and policy implications

In a bid to address undernutrition in the country, the Government of India launched *Poshan Abhiyan* (Nutrition Mission) in early 2018. The mission seeks to scale up existing programs and the Integrated Child Development Services (ICDS)^5^, focusing on key nutrition interventions for improving food intake, dietary diversity, immunization, access to water and sanitation, maternal health indicators among many others. However, as we have noted elsewhere, migrant households continue to face several hurdles in accessing their entitlements, including food and nutrition programs, while in the city [65]. It is paramount that government programs such as these either expand their services or create special provisions to reach migrant communities, who are among the most vulnerable in the country. Our study addresses a critical gap in literature and also elucidates the need to broaden the policy framework to include and address the concerns of migrant households. By focusing on the micro context, this study describes peculiar and specific nutritional challenges and experiences of migrant children at construction sites. The findings of our study may be transferable to similar informal work sites such as brick kilns which see heavy presence of migrant children.

## Data Availability

The data set supporting this article is available with the corresponding author and can be accessed upon reasonable request.

## Footnotes

1 The phrases short-duration, short-term and temporary migration have been used interchangeably in this paper. This includes both seasonal migration, which is defined by National Sample Survey (NSS) as households undertaking migration for 1 month or more up to a period of 6 months and semi-permanent migration, which is defined as those who have been outside of their home for over 6 months.

2 It is estimated that there are approximately 100 million short term migrants in India. Of this, While there is no clear official estimate of the number of migrant children, one of the approximations place them in the range of 10 to 15 million [66].

3 We use the UNICEF definition of undernutrition, which includes underweight, stunting and wasting.

4 The first author can read, write and speak Hindi and has conversational abilities in Gujarati. The research assistant is a native speaker of Gujarati and is also conversant in Hindi. Our knowledge of the two languages enabled us to reduce errors and gaps in translation.

5 The ICDS, which was launched in 1975, is India’s flagship program for improving maternal and child wellbeing by providing nutritious food, health, immunization and preschool education among other services
